# Eculizumab Treatment for Postpartum HELLP Syndrome and aHUS—Case Report

**DOI:** 10.3389/fimmu.2020.00548

**Published:** 2020-04-03

**Authors:** A. Inkeri Lokki, Mikko Haapio, Jenni Heikkinen-Eloranta

**Affiliations:** ^1^Bacteriology and Immunology, University of Helsinki and Helsinki University Hospital, Helsinki, Finland; ^2^Translational Immunology Research Program, Research Programs' Unit, University of Helsinki, Helsinki, Finland; ^3^Obstetrics and Gynecology, University of Helsinki and Helsinki University Hospital, Helsinki, Finland; ^4^Nephrology, University of Helsinki and Helsinki University Hospital, Helsinki, Finland

**Keywords:** case report, preeclampsia, aHUS, eculizumab, HELLP, thrombotic microangiopathy

## Abstract

Preeclampsia is a pregnancy-specific disorder affecting ca 3% of all pregnant women. Preeclampsia is the source of severe pregnancy complications. Later life consequences for mother and infant include increased risk of cardiovascular disease. Preeclampsia is caused by the dysfunction of the endothelium with subsequent activation of complement and coagulation systems. HELLP syndrome is considered to be an extreme complication of preeclampsia but it can also present independently. Diagnostic symptoms in HELLP syndrome are Hemolysis, Elevated Liver enzymes, and Low Platelets. Similar phenotype is present in thrombotic microangiopathies (TMAs) and HELLP syndrome is considered part of the TMA spectrum. Here, we present a case of severe preeclampsia and HELLP syndrome, which exacerbated rapidly and eventually led to need of intensive care, plasma exchange, and hemodialysis. The patient showed signs of hemolysis, disturbance in the coagulation, and organ damage in liver and kidneys. After comprehensive laboratory testing and supportive care, the symptoms did not subside and treatment with complement C5 inhibitor eculizumab was started. Thereafter, the patient started to recover. The patient had pregnancy-induced aHUS. Earlier initiation of eculizumab treatment may potentially shorten and mitigate the disease and hypothetically decrease future health risks of preeclamptic women.

## Introduction

Preeclampsia is a pregnancy-specific disease affecting 3–5% of all pregnancies (1, 2). It manifests with newly onset hypertension after 20 weeks of gestation and proteinuria. The placenta is central in the pathogenesis of the disease by connecting the mother to the fetus (3). The maternal-fetal interface is the zone where genetically different tissue of fetal origin meets with maternal circulation, endothelium, and immune system. Often called the disease of theories, one generally accepted insight is that preeclampsia affects the maternal endothelium causing disturbance in the function of endothelium, which leads to hypertension and proteinuria (2). In its severe form, preeclampsia may lead to fetal growth retardation, prematurity, and for the mother it might cause organ failure in kidneys and liver as well as eclampsia. Furthermore, preeclampsia may have long-term adverse cardiovascular consequences for the mother and the newborn (4). The development of the placenta is considered inadequate specifically in the process of maternal spiral artery transformation, where the trophoblast cells invade the maternal side into decidua and transform the arteries into low resistance conduits, also replacing the maternal endothelium (5–7). Typically, this remodeling of the uterine arteries is absent or incomplete especially in severe form of preeclampsia. High resistance in the constricted uterine arteries causes turbulent blood flow in to the intervillous space of the placenta causing oxidative stress and mechanical damage to the placental villous trees. Resulting damage increases the placental shedding of microparticles and inflammatory mediators resulting in generalized endothelial activation and dysfunction (8, 9). These sequential events in the maternal-fetal interface lead to maternal hypertension and other symptoms as described above.

**Figure 1:**
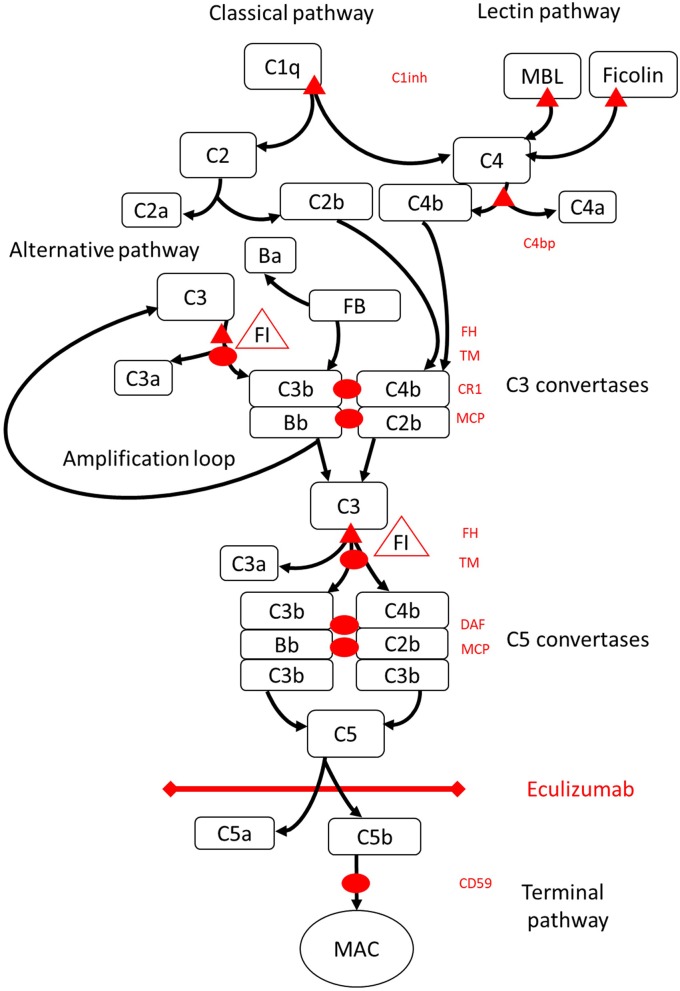
Eculizumab inhibits terminal pathway of complement activation. Complement activation may be initiated via three pathways of activation, that all lead to the formation of C5 convertases that have the capacity to activate the terminal pathway leading to formation of the MAC on the target surface. This schematic illustration of the complement system shows its most relevant activators (in black font) and inhibitors (in red). The activators may be membrane bound (oval) or soluble (triangle). The alternative pathway activates spontaneously on all surfaces that do not allow for inhibition by the soluble regulator factor H (FH). FH acts as the cofactor for inactivation of C3b to iC3b by factor I (FI). Thrombomodulin (TM) enhances FH cofactor activity. Cleaved factor B (FB) together with the activator C3b forms the alternative pathway convertase, which has the capacity to cleave C3 into C3b creating an amplification loop of alternative pathway activation. Classical pathway of complement activation may be initiated by for example binding of immune complexes to C1q, while the lectin pathway is activated by mannoses binding lectin (MBL) or ficolins binding to for example patterns of carbohydrates on microbes. Lectin pathway activation results in mannose-associated serine proteases 1 and 2 (MASP-1 and MASP-2, not pictured) cleaving complement components C4 and C2 to form the classical pathway convertase C4bC2b. C1 inhibitor (C1inh) and C4bp are the soluble regulators of the classical pathway, while membrane cofactor protein (MCP) and complement receptor 1 (CR1) are membrane bound regulators of early complement pathways. The formation of C5 convertases initiates the terminal pathway of complement activation and cleavage of C5 in the absence of surface bound regulators decay accelerating factor (DAF) and MCP. Assembly of MAC is regulated by the surface bound regulator CD59 (protectin). Cleavage of C3 in the early pathways and C5 in terminal pathway releases anaphylatoxins C3a and C5a and results in inflammation. Eculizumab is a humanized recombinant antibody against the complement protein C5, which inhibits cleavage of C5 by the C5 convertases thereby regulating the prothrombotic and proinflammatory effects of complement activation. The patient described in this case report was tested for genetic mutations in genes coding for Factor H (*CFH*), *FHR5*, and *MCP, CFI, CFB, THBD*, and ADAMTS13 (*ADAMTS13*), a regulator of the vWF pathway of coagulation cascade (not shown). The results of the genetic testing were negative.

HELLP syndrome was first characterized in 1982 by Weinstein as a separate syndrome, often representing together with preeclampsia but seen also alone (10, 11). HELLP syndrome is characterized by hemolysis, elevated liver enzymes, and low platelets. It often requires intensive care level observation and symptomatic therapy. The pathogenesis of the syndrome is still in the shadows. HELLP syndrome shares common features with thrombotic microangiopathies (TMAs) such as thrombotic thrombocytopenic purpura (TTP) and hemolytic uremic syndrome (HUS). TMAs present in diverse group of diseases with common features of microangiopathic hemolysis, thrombocytopenia, and organ damage resulting from microthrombi. In TTP, a genetic defect of, or as in vast majority of cases, acquired autoantibodies against a disintegrin and metalloproteinase with thrombospondin type 1 motif 13 (ADAMTS13), the enzyme that cleaves activated von Willebrand factor (vWF), cause formation of unusually large activated vWF multimers on endothelial cells leading to platelet thrombi in small vessels and hemolysis. Typical HUS is caused by Shiga-toxin producing bacterial infection (especially *Escherichia coli*) whereas atypical HUS (aHUS) refers to the type of TMA, in which genetic deficiencies in the regulators of the alternative pathway of complement system underlie, which, in the presence of a trigger may cause the clinical disease (12, 13). The FDA approved treatment for aHUS is the infusion of complement C5 antagonist eculizumab, which prevents the formation of the membrane attack complex (MAC) (Figure 1). Clinically HELLP shares the same symptoms as classical TMAs: hemolysis, thrombocytopenia, and organ disorder seen in liver. Depending on the TMA classification used, HELLP syndrome is usually categorized as part of secondary or acquired TMAs (Table 1).

**Table 1:** HELLP and aHUS diagnostic criteria.

	**HELLP**	**aHUS**
Hemolysis	Plasma haptoglobin below limit for normal, plasma lactate dehydrogenase > 600 U/L	Non-immunological (Coombs test negative) hemolysis with red blood cell fragmentation > 1–2% in peripheral blood smear, plasma haptoglobin below limit for normal, and increased plasma lactate dehydrogenase
Organ dysfunction	Elevated liver enzymes: alanine aminotransferase > 70 U/L	Positive markers of injury (of any organ, but typically acute kidney injury with serum creatinine over 200 μmol/L)
Low platelets	<100 E9/L	Thrombocytopenia (platelet count below 150 E9/L or decrease over 25% from baseline)

In this case study, we report a patient with a complicated pregnancy-induced TMA and the successful course of treatment.

## Case Presentation

Twenty-nine year old primigravida was referred at gestational age 34+2 to the Helsinki University hospital outpatient clinic with upper stomach pain. Initially, her blood pressure was modestly elevated (133/91 mmHg) and urinary dipstick positive for protein. The initially reported upper stomach pain was gradually improving. In the ultrasound scan the fetus had normal biophysical profile (BPP), the weight estimation was at the −2 *SD* growth curve. Cardiotocography (CTG) was normal. Blood hemoglobin (Hb) was 115 g/L, platelets 158 E9/L (normal range 150–360 E9/L), alanine aminotransferase (ALT) was normal (23 U/L). The urinary dipstick was positive for protein (+2) and calculated proteinuria was 1.6 g/24 h. A decision was made to initiate cortisone treatment to facilitate the lung maturation of the baby. The patient was discharged with a plan to return the next day for control check-up and second dose of cortisone. As scheduled, she came for control at gestational week 34+4. Blood pressure was 147/87 mmHg, ALT 23, platelets 177, CTG and the BPP of the fetus in the ultrasound scan was normal. She was discharged and another check-up was scheduled. In the afternoon of the same day, the upper stomach pain returned and steadily worsened toward the evening. She returned to the hospital at 2.20 a.m. She was experiencing tight upper stomach pain, restlessness, and she had vomited two times and was feeling tremor. The blood pressure was clearly elevated at 170/94 mmHg, urine protein dipstick was strongly positive, ALT was elevated at 159, Hb 122, and platelets 172. She was admitted to the prenatal ward. At 4 a.m. she was experiencing headache. Antihypertensive medication was started (Labetalol 100 mg thrice). Urine protein excretion peaked in the night being 13 g/24 h. Subsequently, she started vomiting, had upper stomach pain, headache, and the CTG monitoring showed decelerations. The patient was transferred at 7.11 a.m. to the delivery ward and as the cervix was three centimeters dilatated, the fetal membranes were artificially broken for the induction of labor. At the same time the laboratory tests were completed with Hb 122, platelets 172. Lactate dehydrogenase (LD), however, was clearly elevated at 1231 U/L at this time. In the CTG, the decelerations continued and as bradycardia continued an emergency caesarean section was performed. Male infant (1960 g, −2 *SD*) was born at 7.25 a.m. with umbilical artery pH value of 7.05, BE −6.80, Apgar 1/6/8. Blood loss in the operation was 400 ml.

At 9 a.m. after the caesarean section the mother's platelets were low at 49, with Hb of 102. By the afternoon the ALT had risen to 1800, LD 3570, serum creatinine level was 153 (μmol/L), while platelets decreased to 33. There was disturbance in the coagulation indicated by low level of fibrinogen (1.1 g/L, reference values 2–4 g/L) and high level of D-dimer of fibrin (30.2 mg/L, <0.5 mg/L). There was some bleeding from the caesarean section wound, in which additional sutures were placed. At this time, eight units of platelets were administered. Potassium level rose from 4.7 to 5.6 (mmol/L). Hemolysis was clearly observed. Coombs test was negative. Urine excretion was only 10 ml/h. The laboratory test showed clearly a severe disease with signs of damage to both the kidneys and the liver. In addition, there was disturbance in the coagulation system presenting significant coagulation and marked fibrinolysis simultaneously. Magnesium sulfate infusion was started because of hyperreflexia, which is considered a predictive sign of convulsions, a severe complication of preeclampsia. Intravenous dexamethasone 10 mg was started, and the patient was transferred into intensive care unit (ICU).

Further, laboratory tests were issued for differential diagnostic purposes of other medical emergencies (Table 2). The activity of ADAMTS13 was normal 62% (40–130%), which excludes TTP. Serum complement C3 (0.52 g/L, 0.71–1.41 g/L) and C4 (0.07 g/L, 0.12–0.34 g/L) levels were low. Level of soluble terminal complex of the complement (C5b-9, 971 ng/mL, <366 ng/mL) was elevated on the first postpartum day. Antiphospholipid antibodies were not detected, the infection serology concerning Hepatitis B and C, and HIV was negative. From stool sample, the pathogens causing typical HUS tested negative.

**Table 2:** Timeline of the disease diagnostics and treatment.

**Days**	**Diagnostics**	**Treatment**	**Aim**
Ceasarean section	Basic blood count, C-reactive protein, blood chemical values, hemolysis markers, coagulation factors and descriptive, antiphospholipid antibodies, Coombs test, plasma ADAMTS13 activity, and antinuclear antibodies	Transfer to ICU	To exclude TTP, antiphospholipid syndrome, SLE, and autoimmune hemolytic anemia
Postpartum day 1	Plasma C3 and C4 levels, Complement terminal complex-level, C4A and C4B genetic testing	Plasma exchange	
Postpartum day 2	Hepatitis B and C, HIV, and aHUS genetic tests (Complement system)	Plasma exchange, Hemodialysis	To exclude viral hepatitis as a cause of liver damage
Postpartum day 3	Stool sample testing the pathogens causing typical HUS	Transfer back to Women's Hospital recovery room were observation and symptomatic therapy continued	To exclude typical HUS
Postpartum day 4	Basic laboratory tests concerning hemolysis, liver and kidney function, platelets, and coagulation	Hemodialysis, Transfer to the department of Nephrology, first dose of Eculizumab	Diagnosis of aHUS was placed
Postpartum day 5	Basic laboratory tests concerning hemolysis, liver and kidney function, platelets, and coagulation		
Postpartum day 6	Basic laboratory tests concerning hemolysis, liver and kidney function, platelets, and coagulation	Hemodialysis	

The patient was treated with plasma exchange treatment on first and second postpartum day and was hemodialyzed altogether three times over the course of her treatment (days 2, 4, and 6 postpartum).

**Figure 2:**
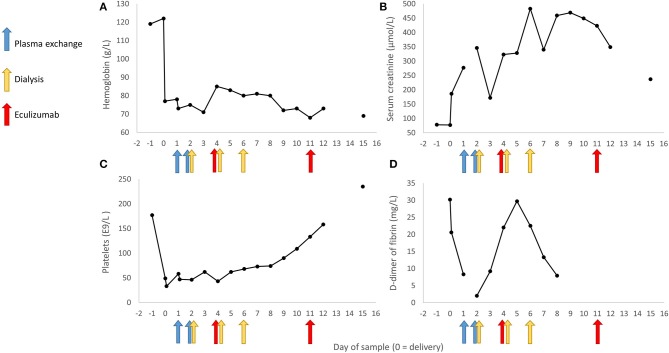
Selected laboratory values observed during the early stages of the disease and the timing of plasma exchange, hemodialysis and administration of eculizumab. In panel **(A)** is represented the development of blood hemoglobin measurements, in panel **(B)** the serum creatinine level, in panel **(C)** the number of platelets, and in panel **(D)** D-dimer of fibrin values across the 17 day follow-up period.

On third postpartum day the patient was stable and transferred back to Women's Hospital recovery room were observation and symptomatic therapy was continued. Hypertension was treated with Amlodipine 10 mg twice a day and Labetalol 200 mg three times a day. On the fourth postpartum day, platelets continued decreasing and the patient was diagnosed with aHUS. Often the differential diagnosis with HELLP syndrome and aHUS lies in spontaneous recovery of HELLP patients usually on third postpartum day. Treatment with eculizumab was started (900 mg IV). Patient received a pneumococcal vaccination and prophylactic antibiotic (penicillin) was started. The patient received all together four weekly doses of eculizumab (900 mg) and she started to recover rapidly. She did not require further hemodialysis after her third hemodialysis on the sixth postpartum day (Figure 2). Kidney function corrected gradually, platelet count elevated, and hemolysis resolved. Four weeks postpartum the plasma levels of C3 and C4 were normalized.

In genetic testing, no known gene polymorphisms were identified. She was tested for mutations in complement regulators Factor H (*CFH*), Factor H related protein 5 (*FHR5*), and membrane co-factor protein (*MCP*), complement alternative pathway inactivator factor I (*CFI*), alternative pathway activator factor B (*CFB*), and the following components of the coagulation cascade: *ADAMTS13*, thrombomodulin (*THBD*), and an intracellular enzyme, diacylglycerol kinase E (*DGKE*), whose mutations are a known causes of aHUS (14). Furthermore, antibodies against factor H were not detected either. No *C4* deficiency was detected.

As a summary, our patient had severe preeclampsia and fulfilled diagnostic criteria for HELLP syndrome. Although genetic testing for aHUS remained negative, the clinical course of the disease (especially, severe acute kidney injury) and response to treatment (especially, eculizumab) suggested that our patient had pregnancy-induced aHUS.

## Discussion

The primigravida described above was diagnosed with severe preeclampsia, HELLP syndrome, and pregnancy-induced aHUS. No known gene mutations, which could predispose to aHUS were discovered. Differential diagnosis between types of TMA, TTP, HUS/aHUS, and secondary TMAs like HELLP syndrome is important. Especially TTP must be identified early on, as the disease is treated with rapid daily plasma exchange until remission (15).

At present, laboratory analysis methods for testing genetic mutations potentially causing aHUS are able to show mutations in only up to 40–60% of aHUS cases, leaving the possibility of false negative cases. Therefore, a negative test result for mutations does not rule out a true aHUS (16). Rarely aHUS has been induced after pregnancy and parturition. In these cases, during the following three years ~50% developed chronic kidney disease (CKD), some even end-stage kidney disease (ESKD). When genetic mutations were observed, up to 85% may develop CKD or ESKD (17).

It is generally accepted that in hypertensive disorders of pregnancy, placental inflammation results in endothelial dysfunction. If the integrity of the endothelium is disturbed this results in activation of complement and coagulation (18). Disrupted maternal endothelium has also been hypothesized to contribute to the later life maternal morbidity associated with severe preeclampsia and other hypertensive pregnancy disorders (2, 19). Damaged endothelium has been shown to function abnormally even years after the initial diagnostic symptoms subside (20–23).

In up to 46% of HELLP patients, gene mutations have been described in the regulators of the alternative pathway of the complement system (24, 25). In the early stages of pregnancy, when the placenta is developing, activation of complement system is seen in elevated levels of Bb in the serum in patients who later developed preeclampsia (26). Activation of the complement system has been observed in severe preeclampsia and HELLP syndrome, and elevated levels of terminal complex (C5b-9) have been detected in urine of patients with severe preeclampsia. In patients with HELLP syndrome, increased activation of complement system was shown by functional test and, furthermore, by deficiencies of expression in CD55 and CD59, leading to decreased regulation and exacerbated activation of the complement system (27–29).

There are many similarities shared between HELLP syndrome and aHUS. In both conditions, endothelium disturbance is clearly involved followed by complement and coagulation activation. In severe preeclampsia and HELLP syndrome, eculizumab given at the early signs of severe disease and TMA would be beneficial for the protection of the kidneys and maternal endothelium (30). Preserving endothelial integrity may potentially protect the patient from long term health risks such as cardiovascular diseases. Typically, in pregnancy-related TMAs, clinical findings of hemolysis, and thrombocytopenia resolve in ~3 days. If disease activity lasts longer, differential diagnostics are to be continued and only then is alternative treatment (e.g., eculizumab) considered. Introducing eculizumab treatment earlier would benefit women by preventing kidney damage and minimizing the turbulence in endothelium and systemic inflammation. Eculizumab, given early enough and as few as one or two doses, could possibly be sufficient to stop the turbulence and be enough to stop the cascade of events (31). On one hand, eculizumab is a very expensive medication, but on the other, the cost of intensive care treatment, plasma exchange, hemodialysis, possible kidney transplantation, not to mention the emotional consequences for the mothers and families remain inestimable.

As a conclusion, our patient was severely ill, experiencing hemolysis, disturbance in the coagulation, liver damage, and kidney failure needing admission to ICU and hemodialysis. It is possible that eculizumab, if initiated earlier, at the first signs of HELLP syndrome, might have been beneficial during the later course of the disease, potentially mitigating kidney injury, and thus preventing need for hemodialysis and later CKD. Therefore, in this era of modern immunological medicine, could we do more for the mothers and families than just wait and hope for the best?

## Ethics Statement

Ethical review and approval was not required for the study on human participants in accordance with the local legislation and institutional requirements. The patients/participants provided their written informed consent to participate in this study.

## Author Contributions

AL and JH-E developed the idea to the manuscript and wrote the manuscript. JH-E and MH were involved in the diagnostic and therapeutic care of the patient. All authors reviewed and edited the manuscript and approved its final version for publication.

### Conflict of Interest

The authors declare that the research was conducted in the absence of any commercial or financial relationships that could be construed as a potential conflict of interest.

## References

[1] Lisonkova S Joseph KS. Incidence of preeclampsia: risk factors and outcomes associated with early-versus late-onset disease. Am J Obstet Gynecol. (2013) 209:544.e1–e12. 10.1016/j.ajog.2013.08.019 23973398

[2] Burton GJ Redman CW Roberts JM Moffett A. Pre-eclampsia: pathophysiology and clinical implications. BMJ. (2019) 366:l2381. 10.1136/bmj.l2381 31307997

[3] Redman CWG Sargent IL. Immunology of pre-eclampsia. Am J Reprod Immunol. (2010) 63:534–43. 10.1111/j.1600-0897.2010.00831.x 20331588

[4] Hauspurg A Ying W Hubel CA Michos ED Ouyang P. Adverse pregnancy outcomes and future maternal cardiovascular disease. Clin Cardiol. (2018) 41:239–46. 10.1002/clc.22887 29446836 PMC6490154

[5] Brosens I Pijnenborg R Vercruysse L Romero R. The “great Obstetrical Syndromes” are associated with disorders of deep placentation. Am J Obstet Gynecol. (2011) 204:193–201. 10.1016/j.ajog.2010.08.009 21094932 PMC3369813

[6] Steegers EA von Dadelszen P Duvekot JJ Pijnenborg R Pre-eclampsia, Seminar. Lancet. (2010) 376:631–44. 10.1016/S0140-6736(10)60279-6 20598363

[7] Ong SS Baker PN Mayhew TM Dunn WR. Remodeling of myometrial radial arteries in preeclampsia. Am J Obstet Gynecol. (2005) 192:572–9. 10.1016/j.ajog.2004.08.015 15696005

[8] Germain SJ Sacks GP Soorana SR Sargent IL Redman CW. Systemic inflammatory priming in normal pregnancy and preeclampsia: the role of circulating syncytiotrophoblast microparticles. J Immunol. (2007) 178:5949–56. 10.4049/jimmunol.178.9.5949 17442979

[9] Tannetta D Masliukaite I Vatish M Redman C Sargent I. Update of syncytiotrophoblast derived extracellular vesicles in normal pregnancy and preeclampsia. J Reprod Immunol. (2017) 119:98–106. 10.1016/j.jri.2016.08.008 27613663

[10] Weinstein L. Syndrome of hemolysis, elevated liver enzymes, and low platelet count: a severe consequence of hypertension in pregnancy. Am J Obstet Gynecol. (1982) 142:159–67. 10.1016/S0002-9378(16)32330-4 7055180

[11] Sibai BM. Diagnosis, controversies, and management of the syndrome of hemolysis, elevated liver enzymes, and low platelet count. Obstet Gynecol. (2004) 103:981–91. 10.1097/01.AOG.0000126245.35811.2a 15121574

[12] George JN Nester CM Syndromes of thrombotic microangiopathy. N Engl J Med. (2014) 371:654–66. 10.1056/NEJMra1312353 25119611

[13] Haller H Thrombotic microangiopathy and the kidneys. Nephrologe. (2019) 14:100–7. 10.1007/s11560-019-0320-4

[14] Brocklebank V Wood KM Kavanagh D. Thrombotic microangiopathy and the kidney. Clin J Am Soc Nephrol. (2018) 13:300–17. 10.2215/CJN.00620117 29042465 PMC5967417

[15] Pourrat O Coudroy R Pierre F. Differentiation between severe HELLP syndrome and thrombotic microangiopathy, thrombotic thrombocytopenic purpura and other imitators. Eur J Obstet Gynecol Reprod Biol. (2015) 189:68–72. 10.1016/j.ejogrb.2015.03.017 25879992

[16] Raina R Krishnappa V Blaha T Kann T Hein W Burke L. Atypical hemolytic-uremic syndrome : an update on pathophysiology, diagnosis, and treatment. Ther Apher Dial. (2019) 23:4–21. 10.1111/1744-9987.12763 30294946

[17] Bruel A Kavanagh D Noris M Delmas Y Wong EKS Bresin E. Hemolytic uremic syndrome in pregnancy and postpartum. Clin J Am Soc Nephrol. (2017) 12:1237–47. 10.2215/CJN.00280117 28596415 PMC5544502

[18] Jokiranta TS. HUS and atypical HUS. Blood. (2017) 129:2847–56. 10.1182/blood-2016-11-709865 28416508 PMC5445567

[19] Paauw ND Lely AT. Cardiovascular sequels during after preeclampsia. In: Kerkhof PLM Miller VM, editors. Sex-Specific Analysis of Cardiovascular Function. Cham: Springer International Publishing (2018). p. 455–70. 10.1007/978-3-319-77932-4_28

[20] Agatisa PK Ness RB Roberts JM Costantino JP Kuller LH McLaughlin MK. Impairment of endothelial function in women with a history of preeclampsia: an indicator of cardiovascular risk. Am J Physiol Hear Circ Physiol. (2004) 286:1–3. 10.1152/ajpheart.00298.2003 15020302

[21] Germain AM Romanik MC Guerra I Solari S Reyes MS Johnson RJ. Endothelial dysfunction: a link among preeclampsia, recurrent pregnancy loss, and future cardiovascular events? Hypertension. (2007) 49:90–5. 10.1161/01.HYP.0000251522.18094.d4 17116761

[22] Kvehaugen AS Dechend R Ramstad HB Troisi R Fugelseth D Staff AC. Endothelial function and circulating biomarkers are disturbed in women and children after preeclampsia. Hypertension. (2011) 58:63–9. 10.1161/HYPERTENSIONAHA.111.172387 21606387

[23] Stanhewicz AE. Residual vascular dysfunction in women with a history of preeclampsia. Am J Physiol Regul Integr Comp Physiol. (2018) 315:R1062–R71. 10.1152/ajpregu.00204.2018 30133302 PMC6734059

[24] Fakhouri F Jablonski M Lepercq J Blouin J Benachi A Hourmant M Factor H, membrane cofactor protein and Factor I mutations in patients with HELLP syndrome. Blood. (2008) 112:4242–545. 10.1182/blood-2008-03-144691 18658028

[25] Vaught AJ Braunstein EM Jasem J Yuan X Makhlin I Eloundou S. Germline mutations in the alternative pathway of complement predispose to HELLP syndrome. JCI insight. (2018) 3:0–13. 10.1172/jci.insight.99128 29563339 PMC5926944

[26] Lynch AM Murphy JR Byers T Gibbs RS Neville MC Giclas PC. Alternative complement pathway activation fragment Bb in early pregnancy as a predictor of preeclampsia. Am J Obstet Gynecol. (2008) 198:385.e1–e9. 10.1016/j.ajog.2007.10.793 18221926 PMC2362503

[27] Burwick RM Velásquez JA Valencia CM Gutiérrez-Marín J Edna-Estrada F Silva JL. Terminal complement activation in preeclampsia. Obstet Gynecol. (2018) 132:1477–85. 10.1097/AOG.0000000000002980 30399106

[28] Vaught AJ Gavriilaki E Hueppchen N Blakemore K Yuan X Seifert SM. Direct evidence of complement activation in HELLP syndrome: a link to atypical hemolytic uremic syndrome. Exp Hematol. (2016) 44:390–8. 10.1016/j.exphem.2016.01.005 26921648 PMC4995062

[29] Sabau L Terriou L Provot F Fourrier F Roumier C Caron C. Are there any additional mechanisms for haemolysis in HELLP syndrome. Thromb Res. (2016) 142:40–3. 10.1016/j.thromres.2016.03.014 27128171

[30] Cofiell R Kukreja A Bedard K Yan Y Mickle AP Ogawa M. Eculizumab reduces complement activation, inflammation, endothelial damage, thrombosis, and renal injury markers in aHUS. Blood. (2015) 125:3253–62. 10.1182/blood-2014-09-600411 25833956 PMC4449039

[31] Elabd H Elkholi M Steinberg L Acharya A. Eculizumab, a novel potential treatment for acute kidney injury associated with preeclampsia/HELLP syndrome. BMJ Case Rep. (2019) 12:e228709. 10.1136/bcr-2018-228709 31492725 PMC6731826

